# Overexpression of Neuron-Derived Orphan Receptor 1 (NOR-1) Rescues Cardiomyocytes from Cell Death and Improves Viability after Doxorubicin Induced Stress

**DOI:** 10.3390/biomedicines9091233

**Published:** 2021-09-16

**Authors:** Per-Christian Berg, Åse Mari Larsen Hansson, Øystein Røsand, Gurdeep Marwarha, Morten Andre Høydal

**Affiliations:** Group of Molecular and Cellular Cardiology, Department of Circulation and Medical Imaging, Faculty of Medicine and Health, Norwegian University of Technology and Science (NTNU), 7030 Trondheim, Norway; perchristberg@gmail.com (P.-C.B.); amhansso@stud.ntnu.no (Å.M.L.H.); oystein.rosand@ntnu.no (Ø.R.); gurdeep.marwarha@ntnu.no (G.M.)

**Keywords:** cardiomyocyte stress, reactive oxygen species, doxorubicin, apoptosis, cell death, cardioprotection

## Abstract

Following myocardial infarction, reperfusion injury (RI) is commonly observed due to the excessive formation of, e.g., reactive oxygen species (ROS). Doxorubicin (DOX), a widely used anti-cancer drug, is also known to cause cardiotoxicity due to excessive ROS production. Exercise training has been shown to protect the heart against both RI- and DOX-induced cardiotoxicity, but the exact mechanism is still unknown. Neuron-derived orphan receptor 1 (NOR-1) is an important exercise-responsive protein in the skeletal muscle which has also been reported to facilitate cellular survival during hypoxia. Therefore, we hypothesized that NOR-1 could protect cardiomyocytes (CMs) against cellular stress induced by DOX. We also hypothesized that NOR-1 is involved in preparing the CMs against a stress situation during nonstimulated conditions by increasing cell viability. To determine the protective effect of NOR-1 in CMs stressed with DOX challenge, we overexpressed NOR-1 in AC16 human CMs treated with 5 µM DOX for 12 h or the respective vehicle control, followed by performing Lactate dehydrogenase (LDH) activity, 3-(4,5-dimethylthiazol-2-yl)-2,5-diphenyltetrazolium bromide (MTT), and caspase-3 activity assays to measure cell death, cell viability, and apoptosis, respectively. In addition, Western blotting analysis was performed to determine the expression of key proteins involved in cardioprotection. We demonstrated that NOR-1 overexpression decreased cell death (*p* < 0.105) and apoptosis (*p* < 0.01) while increasing cell viability (*p* < 0.05) in DOX-treated CMs. We also observed that NOR-1 overexpression increased phosphorylation of extracellular signal-regulated kinase (ERK) (*p* < 0.01) and protein expression levels of B cell lymphoma extra-large (Bcl-xL) (*p* < 0.01). We did not detect any significant changes in phosphorylation of protein kinase B (Akt), glycogen synthase kinase-3β (GSK-3β) and signal transducer and activator of transcription 3 (STAT3) or expression levels of superoxide dismutase 2 (SOD2) and cyclin D1. Furthermore, we demonstrated that NOR-1 overexpression increased the cell viability (*p* < 0.0001) of CMs during nonstimulated conditions without affecting cell death or apoptosis. Our findings indicate that NOR-1 could serve as a potential cardioprotective protein in response to Doxorubicin-induced cellular stress.

## 1. Introduction

Ischemic heart disease (IHD) is the leading cause of death worldwide. Immediately after blockage of the coronary artery, a series of severe biochemical and metabolic changes occur within the myocardium due to the deprivation of oxygen and nutrients. Minimizing the ischemic time by early reperfusion is the most crucial factor in reducing the extent of ischemic injury [1]. Paradoxically, reperfusion of the myocardial tissue can damage the ischemic tissue and cause the death of cardiomyocytes (CMs). This damage is called myocardial reperfusion injury (RI) and there is currently no optimal treatment [2]. Restoration of the blood flow allows the mitochondrial membrane potential to recover, which leads to further mitochondrial Ca^2+^ overload. During ischemia, the mitochondrial permeability transition pores (mPTP) remain closed due to the low pH. However, the increase in pH during reperfusion, combined with the mitochondrial Ca^2+^ overload, results in the opening of mPTP. Prolonged opening of mPTP results in irreversible damage during reperfusion [3]. An important component causing RI is excess production of reactive oxygen species (ROS) during and following reperfusion. Most cells have protective mechanisms to protect against ROS, which include both nonenzymatic and enzymatic antioxidant systems. Superoxidase dismutase (SOD) is an enzyme family which converts superoxide anions to hydrogen peroxide. The enzymes catalase, glutathione peroxidase, and peroxiredoxin then convert the hydrogen peroxide into water and oxygen [4]. ROS production during reperfusion overwhelms the endogenous antioxidant system [5], leading to apoptosis and necrosis of the myocardial tissue [6]. Doxorubicin (DOX) is a widely used anti-cancer drug [7] which has been reported to have severe and sometimes lethal side effects that may cause both short-term and long-term cardiotoxicity [8,9]. The primary mechanism of DOX-induced cardiotoxicity is increased oxidative stress caused by ROS production and lipid peroxidation [10,11,12]. There are currently no effective strategies to reduce the risk of DOX-induced cardiotoxicity.

One strategy proposed to protect the heart against RI and excess ROS production is ischemic preconditioning (IPC). Short periods of ischemia have been reported to protect the heart both before a major myocardial Infarction (MI) [13] and against DOX-induced cardiotoxicity [14]. In this regard, mechanisms previously known to facilitate IPC may be relevant for the protective effect of DOX-induced cellular stress [15]. Phosphorylation of the prosurvival kinases, protein kinase B (Akt) and extracellular signal-regulated kinase (ERK) have been reported to be central components of the protective effects of IPC [16]. These two proteins are part of the reperfusion injury salvage kinase (RISK) pathway [17]. However, acute activation of the RISK pathway is of high importance since chronic activation of Akt leads to maladaptive cardiac hypertrophy, which is also the case for ERK [18,19]. Phosphorylation of Akt during IPC may lead to cardioprotective effects via inhibition of Bad, Bax, and glycogen synthase kinase-3β (GSK-3β), which in the end results in inhibition of mPTP opening [20]. Activation of ERK has also been shown to inhibit BAD via p90 ribosomal S6 kinase (p90RSK or p90S6K). BAD is inhibited by phosphorylation at serine (Ser) 112 by p90RSK, which results in loss of its antagonistic effects over the anti-apoptotic proteins, Bcl-2 and Bcl-xL [21]. ERK can also inhibit GSK-3β by priming it for phosphorylation at Ser9 by p90RSK [22]. Studies have shown that the inactivation of GSK-3β plays a crucial role in myocardial survival in RI [23].

Since early revascularization is still the most effective way of treating MI, preconditioning the heart will delay the initiation of revascularization, which diminishes the effect of early revascularization [24]. Other methods of preparing the heart for an MI and protect against RI are therefore of interest. Physical activity and exercise training have proven to reduce the risk of IHD and cardiovascular diseases (CVD). Exercise training reduces the risk of having a MI but has also been shown to protect the myocardium against RI [25,26,27]. The exact mechanism by which exercise provides this protection is not well understood, but there are some similarities between exercise training and IPC. Previous studies have shown that aerobic exercise protects the heart against RI by activating Akt and inhibit GSK-3β [28]. Exercise training has also been shown to activate the JAK2/STAT3 signaling pathway in rats, protecting the heart against ischemic injury [29]. During intense exercise training, the formation of ROS can lead to oxidative damage to cell components. However, regular exercise training can elevate ROS production to a tolerable level of damage, which in turn upregulates the cellular antioxidant systems [30].

A meta-analysis identified the Nuclear Receptor subfamily 4 group A member 3 (*NR4A3*) as one of the most exercise- and inactivity-responsive genes in skeletal muscle [31]. As an early response gene, *NR4A3* is induced by various physiological stimuli, including growth factors, cytokines, and hormones [32]. Neuron-derived orphan receptor 1 (NOR-1), encoded by the *NR4A3* gene, is a member of the nuclear receptor family of intracellular transcription factors and is considered to be ligand-independent and constitutively active. Earlier studies have shown that overexpression of NOR-1 in rat hearts increased the activity of JAK2/STAT3 after MI, which resulted in reduced infarct size, neutrophil infiltration, and proinflammatory cytokine release [33]. In skeletal muscle, NOR-1 overexpression led to increased mitochondrial DNA copy number and improved oxidative capacity [34]. Additionally, NOR-1 directly increases lipin 1 expression, leading to increased cellular capacity for oxidative metabolism [35]. Lipin 1 has been found to regulate the cytosolic activation of ERK in skeletal muscle [36]. Interestingly, it has also been reported that NOR-1 is involved in the survival response during hypoxia in neuronal cells [37], endothelial cells [38], and vascular smooth muscle cells [39], but the effect on CMs during ROS-induced stress remains unresolved.

The primary aim of this study was to determine the potential protective effect of NOR-1 overexpression against cellular stress induced by DOX in human CMs. The second aim was to evaluate if overexpression of NOR-1 prepares the CMs during nonstimulated conditions against potential stress by increasing cell viability.

## 2. Methods and Materials

### 2.1. Cells and Cell Treatment

In this study, immortalized AC16 CMs (EMD Millipore/Sigma Aldrich, Darmstadt, Germany) were used. AC16 CMs are derived from adult ventricular heart tissue fused with a fibroblast cell line. Both nuclear and mitochondrial DNA are retained from the primary CMs, which provides a good in vitro model to explore signaling pathways and address questions of cardiac biology at the cellular level [40].

CMs (Millipore) were cultured and maintained in Dulbecco’s Modified Eagle’s Medium (DMEM; Thermo Fisher Scientific, Rockford, IL, USA) containing 12.5% fetal bovine serum (FBS; Thermo Fisher Scientific) and 1% Antibiotic Antimycotic Solution (Sigma, Darmstadt, Germany). DMEM with FBS and Antibiotic Antimycotic Solution is hereafter referred to as culture medium. CMs were seeded and subcultured into multiple cell culture plates (100 mm diameter) at a density of 4 × 10^6^ cells per plate to full confluency. Before the transfection, the passage of the cells was between 6 and 9. The cells were cultured in a saturated humidified atmosphere at 37 °C with 5% CO_2_.

### 2.2. Transfection of AC16 CMs

The AC16 CMs were transfected with either the pReceiver-M12 expression-vector (GeneCopoeia, Rockville, MD, USA) encoding the *NR4A3* gene (NOR-1 protein) or the corresponding empty vector (EV) using PolyFect Transfection Reagent (Qiagen, Hilden, Germany) and by employing the reverse transfection technique. The expression vector for NR4A3 was tagged at the N-terminus with the 3xFLAG label.

Before the transfection, a transfection mix for the NR4A3 vector and EV was made by mixing the expression vector with serum- and antibiotic-free DMEM before waiting 5 min and adding the PolyFect Transfection Reagent. The amount of DMEM added was 50 times the amount of vector used, and the amount of transfection reagent was five times the amount of vector. This mix was incubated for 30 min at 37 °C. AC16 CMs from 12 confluent 100 mm plates were trypsinized and collected in two separate 50 mL tubes and pelleted by centrifugation. The two cell pellets were resuspended in culture medium and added to either the NR4A3 vector transfection mix or the EV transfection mix and plated into a total of 16 new 100 mm plates (Nunc™ EasYDish™ Dishes, Thermo Fisher). Each plate contained 900 ng of expression-vector. An amount of 5 mL of culture medium was added to each plate and incubated for six hours at 37 °C. After the six hours of incubation, the conditioned medium was replaced with fresh culture medium and incubated for 24 h at 37 °C. Western blotting confirmed that the cells had translated the NR4A3 expression vector to NOR-1 protein (Supplements Figure S1).

### 2.3. Doxorubicin Treatment

Doxorubicin hydrochloride (DOX; Sigma-Aldrich, St. Louis, MO, USA) was dissolved in sterile water to make a stock solution with a concentration of 1 mM. This was further diluted with culture medium, giving a final concentration of 5 µM. The DOX concentration used in this study was based on a dose–response curve generated earlier in this study. AC16 CMs were subjected to DOX treatment for 12 h with concentrations ranging from 0 to 5 µM. After 12 h, an LDH (*Lactate dehydrogenase*) release assay was performed on the conditioned-medium. The results from the LDH assay showed that 5 µM of DOX resulted in a significant amount of LDH release (Supplement Figure S2).

Four of the 100 mm plates transfected with the NR4A3 expression vector and four of the 100 mm plates transfected with EV were treated with 10 mL culture medium containing 5 µM DOX. A nonstimulated vehicle control group was made by treating the rest of the plates with 10 mL culture medium containing sterile water instead of DOX. All plates were then incubated for 12 h at 37 °C. In addition, two empty plates (negative control with no cells) containing culture medium, with either DOX or sterile water, were incubated. These two plates were used during the LDH assay as negative control blanks.

After 12 h of incubation, the conditioned medium from all 16 transfected plates with CMs was aspirated into separate tubes, later used for the LDH assay. The medium from the two empty plates containing culture medium with either DOX or sterile water was also collected, later used as the negative control blank for the LDH assay. The cells were thereafter washed 2 times with PBS (phosphate-buffered saline) before adding 1 mL of M-PER lysis buffer (Thermo Scientific, Waltham, MA, USA), containing 1% Halt™ Protease Inhibitor Cocktail (Thermo Scientific) and 1% Halt™ Phosphatase Inhibitor Cocktail (Thermo Scientific), was added to each plate to lyse the cells. The plates were incubated on ice for 15 min with repeated intervals of vortexing. The cell lysates were collected into separate 1.5 mL tubes and centrifuged for 15 min at 12,000× *g* and 4 °C. The supernatant representing the cell lysates was transferred to a new set of 1.5 mL tubes and stored at −18 °C. 

### 2.4. Protein Concentration Measurements by Bradford Assay

Bradford assay was performed by using the Pierce Coomassie Plus Bradford Assay Kit (Thermo Scientific). First, a standard was made by diluting 25 µL of Albumin Standard (2 mg/mL) in 1975 µL MilliQ H_2_O to yield a 25 ug/mL. More standards were made through 1:1 serial dilution of the first standard. An amount of 640 µL of each standard was mixed with 160 µL Pierce Coomassie Plus Assay Reagent and plated in triplicates in a 96-well plate. For the negative control blank, 640 µL of MilliQ H_2_O was mixed with 160 µL Pierce Coomassie Plus Assay Reagent and plated in triplicate in a 96-well plate. The dilution series now contained standards with known concentrations ranging from 0 to 4 µg/mL of protein.

For the determination of protein content of the samples, 10 µL of the cell lysate was diluted with 40 µL of M-PER lysis buffer. In total, 4 µL of the diluted sample was mixed with 636 µL MilliQ H_2_OFor the negative control blank, 4 µL M-PER lysis buffer was mixed with 636 µL MilliQ H_2_O. Additionally, 160 µL Pierce Coomassie Plus Assay Reagent was added to the 640 µL of each diluted sample and 200 µL was plated in triplicates in the same 96-well plate as the standards. The absorbance was measured immediately with the FLUOstar Omega microplate reader at 595 nm.

### 2.5. Western Blotting

Cell lysates were diluted to a protein concentration of 5 µg/µL using M-PER lysis buffer. The amount of M-PER added to each sample was based on the protein concentration from the Bradford assay. In total, 200 µL of each sample was then mixed with 200 µL of 2× Laemmli Sample Buffer (Santa Cruz Biotechnology, Dallas, TX, USA), giving the samples a final protein concentration of 2.5 µg/µL.

The samples were loaded into the wells of a 15-well polyacrylamide gel. The amount of sample loaded and the percentage of polyacrylamide in the gels varied based on the protein of interest (Table 1). MagicMark™ XP Western Protein Standard (Invitrogen, Carlsbad, CA, USA) and Precision Plus Protein All Blue Prestained Protein Standard (Bio-Rad, Hercules, CA, USA) were used as protein molecular weight markers. The loaded gels were placed in the electrophoresis chamber and filled with running buffer (25 mM Tris, 192 mM Glycine, 0.1 *w*/*v* SDS). Electrophoresis was performed at 150 mA for 90 min for all the gels.

After electrophoresis, the gels were blotted to PVDF membranes (Immuno-Blot^®^, Bio-Rad), which were pre-wetted in methanol. Blotting was carried out by stacking the gels together with PVDF membranes and placed in a transfer chamber filled with transfer buffer (25 mM Tris, 192 mM Glycine, pH 8.1–8.5). The transfer was run at 200 mA for 90 min for all proteins. Following the transfer, the membranes were washed three times with TBS-T for a total of 15 min before blocking with 5% nonfat dried milk in TBS-T for 60 min at room temperature. The membranes were then rewashed with TBS-T for 15 min before adding the primary antibody. This was achieved by diluting the primary antibodies with 5 mL TBS-T for each membrane. Table 2 shows the amounts and dilutions of the primary and secondary antibodies used for the respective membranes. The membranes with the primary antibody were incubated overnight at 4 °C. After incubation with the primary antibody overnight, the membranes were washed with TBS-T for 30 min and incubated with the secondary antibody for 90 min at room temperature, followed by washing with TBS-T for 60 min. The membranes were then put into a 1:1 mix of SuperSignal West Pico PLUS Luminol/Enhancer Solution (Thermo Scientific) and SuperSignal West Pico PLUS Stable Peroxide Solution (Thermo Scientific) for one minute and were then imaged using the LI-COR Odyssey FC (LI-COR, Lincoln, NE, USA). Quantification was carried out with the LI-COR Image Studio Software.

### 2.6. LDH Assay

The CytoTox 96^®^ Non-Radioactive Cytotoxicity Assay kit (Promega, Madison, WI, USA) was used to determine the LDH release from the cells. This assay was performed immediately after collecting the conditioned-medium during the cell harvest. In total, 200 µL conditioned medium from each tube was diluted with 800 µL phosphate-buffered saline (PBS). Then, 50 µL of the diluted conditioned medium was added to a well in a 96-well plate, and each biological replicate had three technical replicates. Additionally, 50 µL of CytoTox 96^®^ Reagent was added to each well, and the plate was incubated at room temperature, in the dark, for 60 min. An amount of 50 µL of stop solution was then added to each well, and the absorbance was immediately measured at 490 nm using the FLUOstar Omega (BMG LABTECH, Ortenberg, Germany) microplate reader.

### 2.7. MTT Assay

Cell viability was determined using the MTT assay (Cell Proliferation Kit I, Sigma Aldrich/Roche). The MTT assay was performed in a 96-well plate. CMs were transfected as previously described but seeded at a density of 3 × 10^4^ cells per well. Two rows, 24 wells, were seeded with CMs containing the NR4A3 vector transfection mix, and two rows were seeded with CMs containing the EV transfection mix. Each well now contained 9 ng of the expression vector. The culture medium was changed after 6 h and incubated for 24 h. After this, the medium in half of the wells containing NR4A3 vector and EV was changed to culture medium containing 5 µM DOX. The other half received culture medium with sterile water instead of DOX. After 12 h of incubation, the medium was aspirated and replaced with 100 µL colorless DMEM (DMEM without the phenol red dye) and 10 µL MTT Reagent. Three empty wells (without cells but containing the 100 µL colorless DMEM) also received this to serve as negative control blanks. Two hours later, 100 µL of Solubilization buffer was added to each well to solubilize the formed formazan crystals. The absorbance levels in each well were determined at 570 nm using the FLUOstar Omega microplate reader.

### 2.8. Caspase-3 Assay

AC16 cells were transfected and treated with DOX as previously described. After the DOX treatment, the conditioned-medium from all 16 plates was aspirated, and 1 mL of trypsin was added to each plate. Following trypsinization, the cells were pelleted in separate 1.5 mL tubes by centrifugation at 500× *g* for 5 min. The cell pellets were resuspended in 250 µL lysis buffer (50 mM HEPES, 5 mM CHAPS, pH 7.4). This cell suspension was centrifuged at 12,000× *g* for 15 min at 4 °C. The supernatant containing the cell lysates was collected in a new set of tubes and incubated on ice while the protein concentration was determined by Bradford assay. Following the Bradford assay, 200 µL of each cell lysate was diluted to a concentration of 5 µg/µL, and 60 µL of the cell lysate was added to a well in a 96-well plate in triplicates. To each well, 120 µL of assay buffer (20 mM HEPES, 1.62 mM CHAPS, 10 mM NaCl, 2 mM EDTA, pH 7.4) and 20 µL caspase-3 substrate (Merck, Darmstadt, Germany) were added. A negative control blank (no cells) was made by mixing 60 µL of lysis buffer with 120 µL of assay buffer and 20 µL caspase-3 substrate. The absorbance levels in each well were determined at 405 nm using the FLUOstar Omega microplate reader. List of all reagents used could be found in Table S1.

### 2.9. Statistical Analysis

Data are presented as means with SD. Testing for normality was carried out by using Shapiro–Wilk test. Differences between NR4A3 vector and empty vector in the control- and DOX group were analyzed using unpaired multiple t-tests corrected for multiple comparisons using the Holm–Sidak method. The statistical analyses were performed in GraphPad Prism (version 9.1.2). *p* < 0.05 was considered significant.

## 3. Results

**Figure 1:**
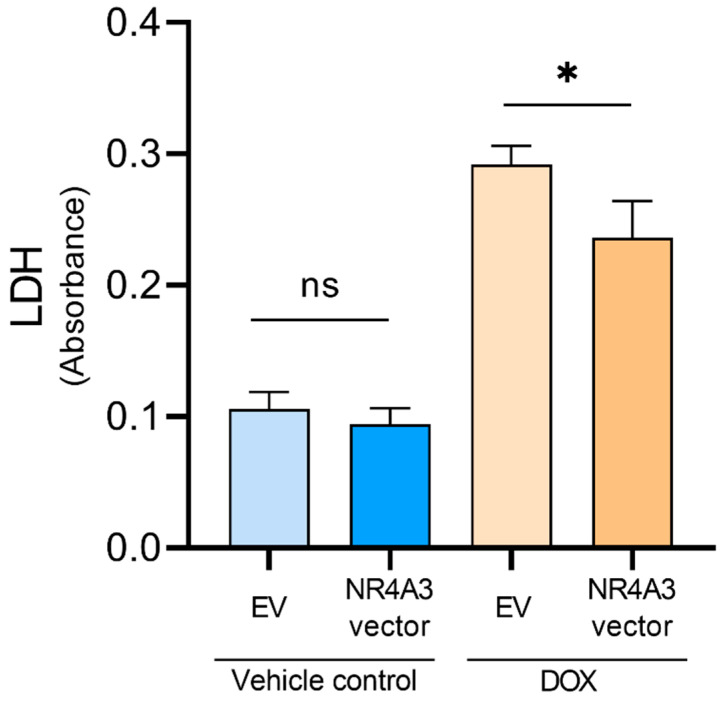
Effect of NOR-1 overexpression on cell death measured by LDH release in DOX-treated CMs. CMs were transfected with expression vectors encoding NR4A3 or the respective empty vector (EV) before treatment with 5 µM of DOX for 12 h. The extent of cell death was determined by measuring LDH release in the condition medium after DOX treatment. Absorbance was measured at 490 nm. Biological replicates in each group (*N* = 4), with three technical replicates for each biological replicate. The graph is presented with mean ± SD. ns = *p* > 0.05 (not significant), * = *p* ≤ 0.05.

To determine if overexpression of NOR-1 could protect the cells against DOX-induced stress, we performed an LDH release assay. First, we confirmed that treatment of the CMs with 5 µM of DOX for 12 h increased the LDH release within the EV control-treated groups (Figure 1). In NOR-1 overexpressing CMs, we found that DOX-induced LDH release was significantly reduced compared to EV CMs (*p* < 0.05). NOR-1 overexpression did not affect LDH release in unstimulated vehicle-treated cells.

**Figure 2:**
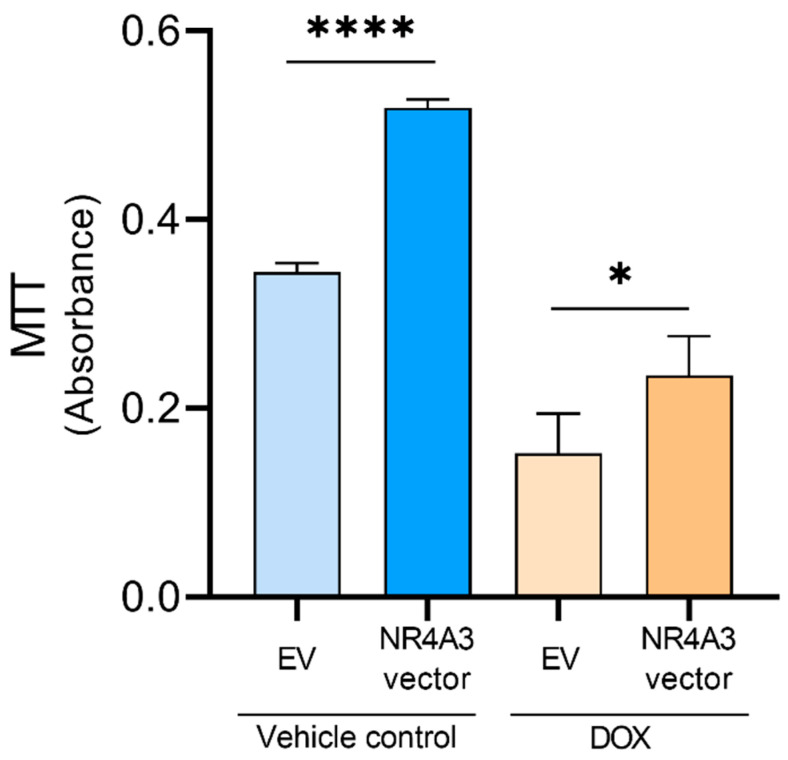
Effect of NOR-1 overexpression on cell viability in DOX-treated CMs measured by MTT assay. CMs were seeded at 3 × 10^4^ cells per well in a 96-well plate and transfected with 9 ng of vector per well. Biological replicates in each group (*N* = 4), with three technical replicates for each biological replicate, giving 12 wells for each treatment group. Absorbance was measured at 570 nm. The graph is presented with mean ± SD. * = *p* ≤ 0.05, **** = *p* ≤ 0.0001.

To determine whether the overexpression of NOR-1 would impact on cell viability, both in nonstimulated conditions and under DOX-induced stress, we performed an MTT assay. We found that DOX treatment led to decreased CMs viability within the EV-transfected groups (Figure 2). NOR-1 overexpression increased CM viability in both DOX-treated CMs (*p* < 0.05) and in the unstimulated vehicle-treated CMs (*p* < 0.0001). These results indicate that NOR-1 promotes the survival of DOX-treated CMs.

**Figure 3:**
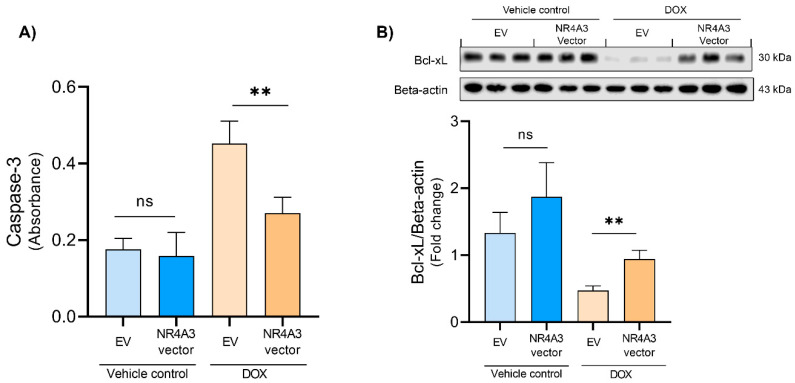
Effect of NOR-1 overexpression on caspase-3 activity and Bcl-xL expression in DOX-treated CMs. (**A**) CMs were transfected with vectors encoding NR4A3 or the corresponding empty vector before treatment with 5 µM of DOX for 12 h. Caspase-3 activity assay was performed after treatment to determine the amount of apoptosis in each group. Absorbance was measured at 405 nm. Each treatment group consisted of four biological replicates and the caspase-3 activity assay was performed on three technical replicates from each biological replicate. (**B**) Western blot showing NOR-1 attenuating the effect DOX treatment on the expression of Bcl-xL normalized to loading control Beta-actin (*N* = 3). The graphs in (**A**,**B**) are presented with mean ± SD. ns = *p* > 0.05 (not significant), ** = *p* ≤ 0.01.

Caspase-3 activation is one of the final mediators in the apoptotic pathway [41]. Therefore, we tested if apoptosis is responsible for the DOX-induced CM death by performing a caspase-3 activity assay. We found that DOX treatment in EV CMs increased caspase-3 activity (Figure 3A). Overexpression of NOR-1 induced a significant reduction in caspase-3 activity compared to EV CMs following DOX stress stimulation (*p* < 0.01). In nonstimulated vehicle control conditions, overexpression of NOR-1 did not have any influence on the already low caspase-3 activity.

Cytochrome c release from the mitochondria is an upstream mediator of caspase-3 activation, and the anti-apoptotic protein Bcl-xL can block the cytochrome c release into the cytosol [42]. Therefore, we measured the protein expression levels of Bcl-xL (Figure 3B). We found that DOX treatment decreased the expression of Bcl-xL, but overexpression of NOR-1 displayed a significantly higher expression of Bcl-xL than the EV-transfected cells (*p* < 0.01). The results from the caspase-3 assay and the changes in protein expression levels of Bcl-xL indicate that apoptosis is a significant cause of cell death in DOX-treated CMs and that NOR-1 inhibits the apoptotic pathway.

**Figure 4:**
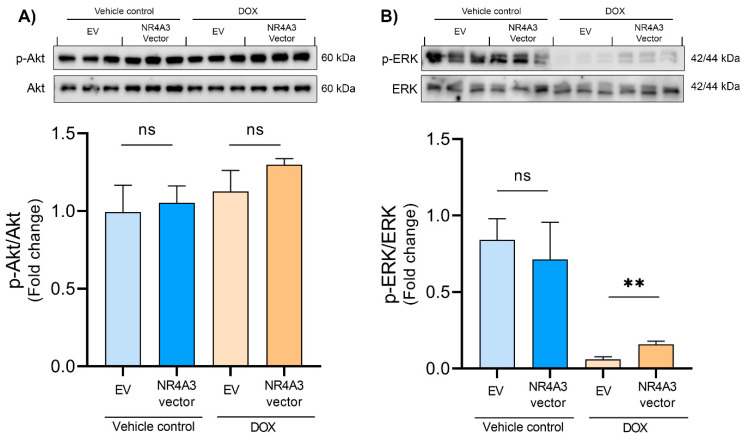
Phosphorylation of Akt and ERK measured by Western blotting. (**A**) Treating the CMs with DOX for 12 h showed no effect on phosphorylation of Akt (Ser473) and neither did transfecting with NR4A3. (**B**) DOX treatment caused a great decrease in phosphorylation of ERK (Thr202/Tyr204). The decrease in phosphorylation was significantly attenuated by transfecting with NR4A3 before DOX treatment. The graphs in (**A**,**B**) are presented with mean ± SD (*N* = 3). ns = *p* > 0.05 (not significant), ** = *p* ≤ 0.01.

Data from the LDH-MTT and caspase-3 assays show that NOR-1 protects the CMs against DOX-induced stress. To further determine the underlying signaling processes involved in the protective effect of NOR-1, we assessed the protein regulation of several key candidates previously reported to enhance resistance against cardiac stress. The first protein of interest was Akt, which is a part of the RISK pathway [43]. Akt is a prosurvival kinase, and its phosphorylation at Ser473 is involved in the cardioprotective effect of ischemic preconditioning [44]. Additionally, DOX has been shown to inhibit phosphorylation of Akt in a concentration- and time-dependent manner in H9C2 CMs [45]. However, in the present study, we did not observe any effect of DOX on Ser473 phosphorylation of Akt in CMs (Figure 4A). Furthermore, NOR-1 overexpression did not affect Akt phosphorylation either.

Another prosurvival kinase in the RISK pathway is ERK [43]. Earlier studies have shown that NOR-1 directly regulates the transcription of lipin 1, which is suggested to play a role in regulating cytosolic activation of ERK [35,36]. Additionally, activation of ERK has been shown to protect CMs against oxidative stress and DOX-induced apoptosis [46,47]. We found that treating the CMs with DOX decreased ERK phosphorylation on Thr202/Tyr204 (Figure 4B). Interestingly, we found an almost three-fold increase in phosphorylation of ERK in the DOX-treated CMs transfected with NR4A3 versus the EV- transfected CMs (*p* < 0.01).

**Figure 5:**
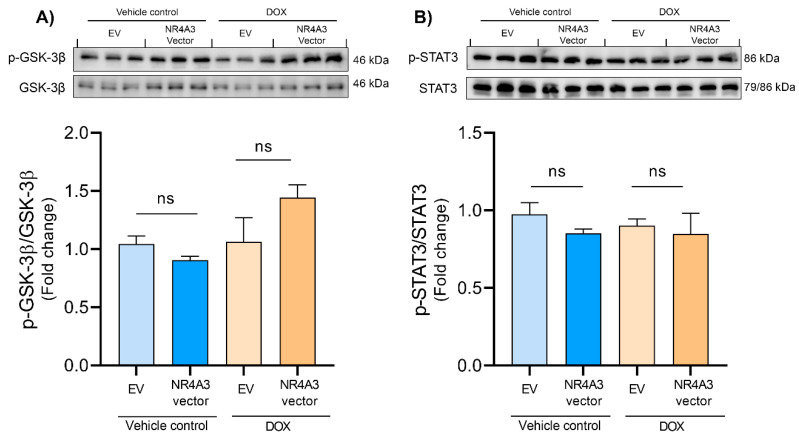
The levels of phosphorylation of GSK-3β and STAT3 measured by Western blotting. The results in (**A**,**B**) show that treating the CMs with 5 µM DOX for 12 h showed no effect on phosphorylation of GSK-3β (Ser9) and STAT3 (Ser727), and neither on NOR-1 overexpression; the graphs in (**A**,**B**) are shown as mean with SD (*N* = 3). ns = *p* > 0.05 (not significant).

Furthermore, to evaluate the downstream targets of ERK, we assessed the phosphorylation of GSK-3β. ERK directly phosphorylates p90RSK, which in turn could inhibit GSK-3β through phosphorylation on Ser9 [48,49]. Our study found that NOR-1 overexpression showed a tendency to modulate the phosphorylation of GSK-3β on Ser9 in the DOX-treated CMs compared to the EV control, but it was not significant (*p* = 0.068, Figure 5A). Furthermore, overexpression of NOR-1 did not significantly alter the phosphorylation of GSK-3β in the nonstimulated vehicle-treated control group either.

Another protein of interest was STAT3. STAT3 plays a part in the survivor activating factor enhancement pathway, and ERK is thought to be involved in the phosphorylation of STAT3 on Ser727 [50,51]. However, we did not observe any significant changes in phosphorylation of STAT3 on Ser727 in this study (Figure 5B).

**Figure 6:**
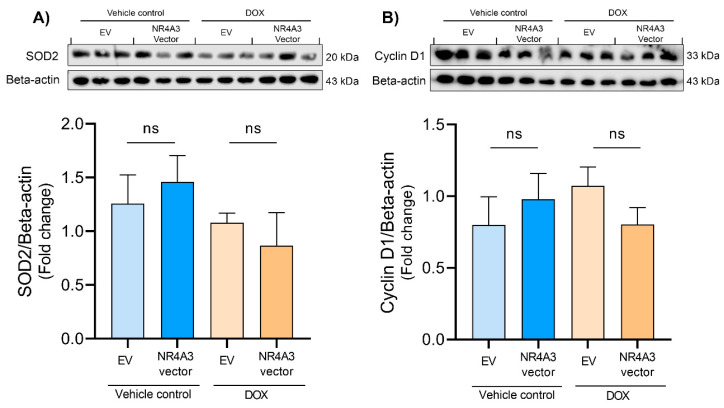
Protein expression of SOD2 and Cyclin D1 measured by Western blotting. The results in (**A**,**B**) show that treating the CMs with 5 µM DOX for 12 h showed no effect on protein expression of SOD2 and Cyclin D1, and neither did NOR-1 overexpression. Protein expression was normalized to loading control Beta-actin in the densiometric analyses. The graphs in (**A**,**B**) are presented with mean ± SD (*N* = 3). ns = *p* > 0.05 (not significant).

The increase in ROS production caused by DOX is known to cause cardiotoxicity, and SOD enzymes control ROS levels in the cells [52,53]. Therefore, we measured the protein expression of SOD2. However, we did not observe any significant changes in SOD2 expression after NR4A3 transfection (Figure 6A).

We also determined the expression of cyclin D1. Inhibition of GSK-3β on Ser9 causes the induction of cyclin D1, which plays a vital role in cell proliferation [54,55]. In our study, no significant change in the expression of cyclin D1 was observed between the different groups (Figure 6B).

**Figure 7:**
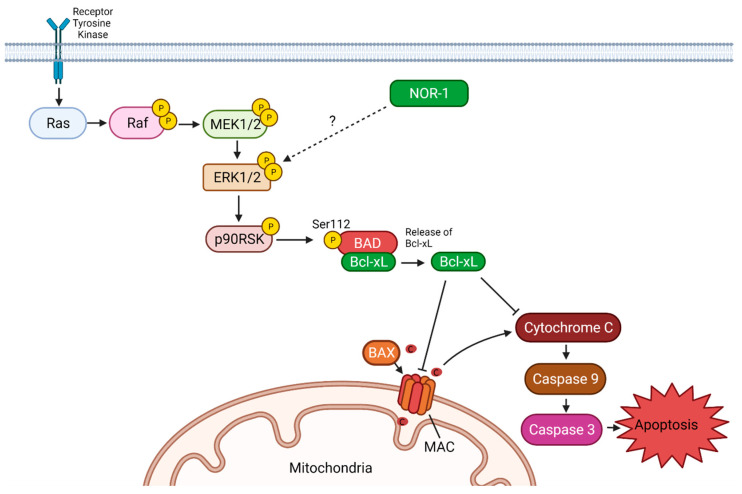
Proposed protective mechanism of NOR-1 overexpression in DOX-treated AC16 cardiomyocytes (CMs). Neuron-derived orphan receptor 1 (NOR-1) overexpression in DOX-treated AC16 CMs led to increased phosphorylation of extracellular signal-regulated kinase 1/2 (ERK1/2). The interaction between NOR-1 and ERK1/2 is currently unknown. ERK1/2 can, through p90 ribosomal S6 kinase (RSK), increase the amount of B cell lymphoma-extra-large (Bcl-xL) [21,22,23,24,25,26,27,28,29,30,31,32,33,34,35,36,37,38,39,40,41,42,43,44,45,46,47,48,49,50,51,52,53,54,55,56]. Bcl-xl inhibits cytochrome c release from the mitochondria by preventing the formation of the mitochondrial apoptosis-induced channel (MAC) and inhibiting cytochrome c in the cytosol [42]. In the end, this results in decreased caspase-3 activity and less apoptosis. Bax, Bcl-2-associated protein X; Bad, Bcl-2-associated death promoter; Ras, rat sarcoma; Raf, rapidly accelerated fibrosarcoma; MEK1/2, Mitogen-activated protein kinase kinase 1/2; Created at 12 May 2021 with BioRender.com.

The mechanism design of the entire study can refer to Figure 7.

## 4. Discussion

Further insight into mechanisms involved in cardioprotective measures against RI after an MI is essential to reduce mortality. The present study explored the effects of NOR-1 overexpression on cell death, cell viability, and apoptosis in CMs. Oxidative damage to the ischemic tissue caused by ROS has gained support as one of the primary mechanisms to explain the cellular damage caused by RI [57]. Excessive ROS production has also been proposed as the primary mechanism in which DOX injures the myocardium [58]. Therefore, we treated the CMs with 5 µM DOX for 12 h to induce oxidative stress. In parallel, we explored the effects of NOR-1 overexpression in a nonstimulated vehicle control group. Following this, we investigated important signaling pathways earlier reported to facilitate cardioprotection.

To our knowledge, there are no previous studies NOR-1 in AC16 CMs. There are, however, several studies that determined the effect of DOX treatment in AC16 CMs [59,60,61]. Yuan et al. treated AC16 CMs with DOX and observed that cell death occurred in a time- and dose-dependent manner [59]. In the initial parts of the present study, we evaluated the dose-response curve of DOX in AC16 CMs and found that 5 µM of DOX for 12 h increased the LDH release significantly. This treatment gave us a good model to explore the possible protective effects of NOR-1 overexpression against DOX-induced cellular stress.

Overexpression of NOR-1 decreased the amount of LDH release in DOX-treated CMs, indicating reduced cell death. However, the increased LDH release in the culture medium only means that the plasma membrane is damaged and does not indicate if the cell death occurred by apoptosis, necrosis, or another form of cellular damage [62]. In our nonstimulated vehicle control group, overexpression of NOR-1 did not influence the LDH release. The activity of NOR-1, and other members of the subfamily of NR4A orphan nuclear receptors, is regulated by expression levels, protein-protein interactions, and posttranslational modifications [63]. Our results indicate that the increased protein level of NOR-1 does not affect cell death in a nonstimulated condition but is involved in protecting the CMs during DOX-induced stress.

Caspase-3 activation is a strong indication of apoptotic cell death [64]. The caspase-3 activity assay indicated that DOX-treated CMs underwent apoptotic cell death, which was also observed by Pan et al. [65]. Furthermore, the caspase-3 activity assay showed that NOR-1 is a potent inhibitor of apoptosis in DOX-treated CMs. NOR-1 overexpression of the CMs resulted in significantly less caspase-3 activity compared to the EV-treated CMs. These data support the results from the LDH activity assay, indicating that NOR-1 plays a major role in protecting the CMs through the apoptotic pathway during DOX-induced stress. Furthermore, NOR-1 overexpression in a nonstimulated condition did not alter the amount of caspase-3 activity significantly. This finding further strengthens our observations in the LDH assay that NOR-1 overexpression does not influence cell death during nonstimulated conditions.

We determined cell viability by MTT assay and observed decreased cell viability between the EV-CMs in the vehicle control and DOX-treated groups. Chen et al. also observed reduced cell viability when treating CMs with 5 µM DOX for 24 h [60]. In our DOX-treated CMs, NOR-1 overexpression improved cell viability significantly, indicating that NOR-1 plays a positive role in reducing cell death during DOX-induced stress. Interestingly, NOR-1 overexpression also increased the cell viability in the nonstimulated vehicle control group. These findings could support our hypothesis that NOR-1 overexpression increases cell viability, which prepares the CMs for an eventual stress condition. In addition, NOR-1 overexpression did not alter apoptosis or cell death under nonstimulated conditions. Furthermore, given that NR4A3 is identified as one of the most exercise-responsive genes in skeletal muscle [31] and that exercise training protects the heart during ischemia and reperfusion [66] this could serve as a link between the cardioprotective effects seen after exercise training and NOR-1. The attenuation of cell death could be explained by both increased BCL-xL and pERK1/2 induced by increased NOR-1 expression in DOX stressed cells (Figure 7). However, none of these were changed in the vehicle control cells and could therefore not explain the increased viability measured by MTT in these conditions. Further studies are therefore needed to determine the exact mechanisms.

Having observed that overexpression of NOR-1 protected the CMs against DOX-induced stress, the aim was to further explore the underlying signaling processes involved in the protective effect of NOR-1. Given that many of the factors that cause RI have also been implicated as mediators of DOX cardiotoxicity [15], and the recent finding that IPC was cardioprotective against DOX-induced cardiotoxicity [67], we examined the protein expression of key prosurvival kinases involved in the RISK pathway [43]. According to several studies, Akt phosphorylation is an essential factor in cardioprotection during MI after IPC [68,69]. DOX treatment has been reported to reduce the amount of Akt phosphorylation in rat hearts and the H9C2 cell line derived from rat heart tissue [70,71,72]. Surprisingly, in our study, DOX treatment did not decrease Akt phosphorylation. One possible reason for this might be the duration of DOX treatment. In the study on the H9C2 cell line, the duration of DOX treatment lasted for 24 h, compared to 12 h in the present study [70]. Thus, a longer treatment time might be needed to modulate the phosphorylation of Akt in CMs. However, our findings do not rule out the possibility for NOR-1 to phosphorylate Akt as a stress response.

Chen et al. found that both Akt and ERK phosphorylation protected CMs against DOX-induced apoptosis [47]. In the present study, we observed that DOX decreased ERK phosphorylation and NOR-1 overexpression significantly attenuated this effect. ERK is involved in the RISK pathway and exerts anti-apoptotic functions in CMs during stress [19]. In addition, ERK inhibits apoptosis by preventing caspase-8 and caspase-9 activation in other cell types [73,74] and could thus explain the observed effect of NOR-1 overexpression on caspase-3 activity in our study.

Furthermore, ERK can also inactivate the proapoptotic protein BAD via p90RSK [56], which causes loss of the inhibitory effect of BAD on Bcl-xL [21]. Our study observed that NOR-1 overexpression significantly increased the already low protein expression of Bcl-xL in CMs after DOX treatment. This finding could explain the decrease in apoptosis observed in DOX-treated CMs by overexpression of NOR-1. Bcl-xL prevents cytochrome c release from the mitochondria, resulting in inhibition of apoptosis. Another way Bcl-xL can inhibit apoptosis is by binding to cytochrome c in the cytosol and thereby preventing the formation of the apoptosome complex [42].

Furthermore, ERK also targets and inhibits GSK-3β [22]. A protective component of GSK-3β inhibition during myocardial ischemia and reperfusion is to increase the threshold for mPTP opening [75] as well as prevent BAX from translocating to the mitochondrial membrane [76]. Furthermore, GSK-3β inactivation in other cell types leads to an accumulation of β-catenin in the cytoplasm. The accumulated β-catenin then translocates to the nucleus and modulates the transcription of genes such as cyclin D1 and c-myc [22]. Hanh et al. overexpressed β-catenin in both CMs and cardiac fibroblasts and observed decreased apoptosis after an MI. The possible protective effects of β-catenin were through upregulation of Bcl-2, survivin, cyclin D1, and cyclin E2 [77]. However, overexpression of NOR-1 did not seem to significantly modulate the protein levels of phosphorylated GSK-3β nor cyclin D1 in our DOX-treated CMs (Figure 5A and Figure 6B). The NOR-1 overexpression showed a tendency cause phosphorylation of GSK-3β during DOX-induced stress, but we cannot draw any conclusions from this. Further studies are therefore needed to explore if NOR-1 overexpression alters GSK-3β phosphorylation. ERK can also phosphorylate STAT3 at Ser727 [50], but we observed no significant changes in phosphorylated STAT3 levels in our study.

Excessive ROS production has gained support as the primary cause in which DOX induces cardiotoxicity [78], and one of the scavengers of mitochondrial ROS is SOD2 [79]. Chaiswing et al. demonstrated that SOD2 overexpression in mice protected against DOX-induced cardiotoxicity [80]. Cheung et al. showed that DOX treatment of H9C2 CMs resulted in a dose-dependent decrease in SOD2 expression [81]. Surprisingly, DOX treatment did not alter the protein expression of SOD2 in our study. Like Akt, a longer treatment time might be needed to alter SOD2 expression. Furthermore, exercise training increases the expression levels of SOD1 in cardiac tissue after exercise training and might play a part in protection against DOX-induced damage [82]. One interesting finding by Alonso et al. when using vascular smooth muscle cells was that NOR-1 overexpression indirectly downregulated SOD2 [83]. However, we did not observe any significant changes in SOD2 expression levels after NOR-1 overexpression. Our results could indicate that NOR-1 overexpression does not initially protect the CMs against DOX-induced stress through modulating SOD2 expression levels. However, the effect of NOR-1 on other enzymatic antioxidants should not be ruled out.

## 5. Conclusions

The present study has given new insights into the potential protective effects of NOR-1 overexpression in CMs. We demonstrated that NOR-1 overexpression decreased cell death and apoptosis and increased cell viability in DOX-treated CMs. This protective effect of NOR-1 overexpression involves the phosphorylation of ERK and increased levels of Bcl-xL. Furthermore, we demonstrated that NOR-1 overexpression increased the cell viability of CMs during nonstimulated conditions without affecting cell death and apoptosis. Thus, our findings indicate that NOR-1 could serve as a novel candidate for cardioprotection against DOX-induced cellular stress. The observations in the present study may serve as a platform for further studies to evaluate the clinical potential for NOR-1 as treatment against ROS-induced cellular stress during RI, as well as against DOX-induced cardiotoxicity seen as a severe side effect when DOX are used during anti-cancer treatment.

## 6. Limitations

Despite the fact that DOX stress share many of the same factors causing injury to the heart as IR, it cannot compare to the complete scenario occurring during an MI. In this regard, it is important to note that the present study only assesses the effect of NOR-1 and DOX in an in vitro model, and the effects observed may be different in vivo. Furthermore, the use of a cell line (human AC16 CMs) should also be taken into consideration as cell lines do not entirely mimic primary cells [84]. However, it if difficult to maintain primary human cardiac cells in cell culture and this requires internal organ biopsy [85]. When comparing to primary cells from animal models, the AC-16 CMs provide a model that is easily genetically manipulated, yields higher biological sample volume, and, importantly, also optimal genomic specificity for translation to human applications [40]. The current project only determined the effect of NOR-1 using one concentration and transfection time. Ideally, the effect of both different concentrations and treatment times with NOR-1 overexpression, as well as DOX, could also have provided valuable information. Therefore, our work should, in the future, be repeated using primary cardiac cells to validate our findings further. The present data also show promise for further studies to test the effect of NOR-1 against cardiac DOX-induced stress responses as well as IR injury in vivo.

## Figures and Tables

**Table 1:** Amount of protein loaded, and gel percentage used during Western blotting.

Protein of Interest	Amount Protein Loaded (µg)	Gel Percentage (%)
Beta-actin	30	10
NOR-1	40	10
Phospho-Akt (Ser473)	40	10
Akt	20	10
Phospho-ERK1/2 (Thr202/Tyr204)	40	10
ERK1/2	20	10
Phospho-GSK-3β (Ser9)	50	10
GSK-3β	20	10
Phospho-STAT3 (Ser727)	60	10
STAT3	20	10
Bcl-xL	40	12
SOD2	50	12
Cyclin D1	30	10

**Table 2:** Dilutions used for the different primary and secondary antibodies.

Proteins of Interest	Host	Primary Antibody Dilution in 5 mL TBS-T	Secondary Antibody Dilution in 10 mL TBS-T
Beta-Actin	Mouse	1:1000	1:3000
NOR-1	Mouse	1:500	1:3000
Phospho-Akt (Ser473)	Rabbit	1:1000	1:2000
Akt	Rabbit	1:1000	1:2000
Phospho-ERK1/2 (Thr202/Tyr204)	Rabbit	1:1000	1:2000
ERK1/2	Rabbit	1:1000	1:2000
Phospho-GSK-3β (Ser9)	Rabbit	1:1000	1:2000
GSK-3β	Rabbit	1:1000	1:2000
Phospho-STAT3 (Ser727)	Rabbit	1:1000	1:2000
STAT3	Mouse	1:1000	1:3000
Bcl-xL	Rabbit	1:2000	1:2000
SOD2	Rabbit	1:1000	1:2000
Cyclin D1	Rabbit	1:300	1:2000

## Data Availability

All data included in the manuscript.

## Supplementary Materials

The following are available online at https://www.mdpi.com/article/10.3390/biomedicines9091233/s1, Figure S1: NOR-1 expression—representative western blots and densitometric analysis; Figure S2: Dose-response curve of DOX induced cell death in AC16 cardiomyocytes (CMs) determined by LDH assay, Table S1: List of reagents used.
