# Supplementary material for: Overexpression of Neuron-Derived Orphan Receptor 1 (NOR-1) Rescues Cardiomyocytes from Cell Death and Improves Viability after Doxorubicin Induced Stress
Source: Biomedicines. 2021 Sep 16;9(9):1233. doi: 10.3390/biomedicines9091233 (PMC8471245; doi:10.3390/biomedicines9091233)
Supplement: Supplementary file 1 [file biomedicines-09-01233-s001.zip › biomedicines-1374525-supplementary.pdf]

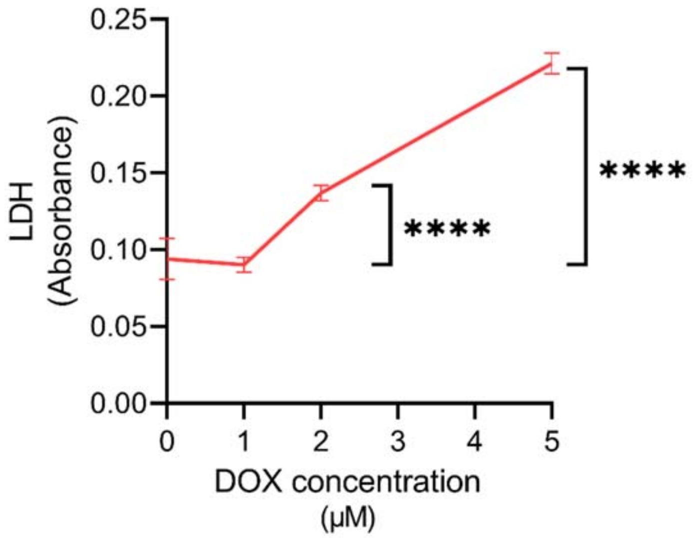

Supplement Figure S1: Validation of NOR-1 overexpression**.** Western blotting validating NOR-1 overexpression in the transfected CMs. mean ± SD. **** = $p \leq 0.0001$.

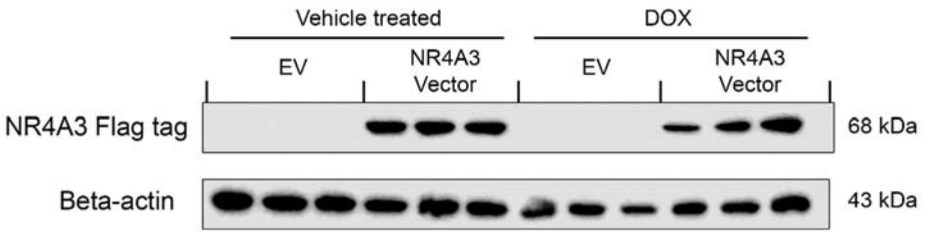

Supplement Figure S2: Dose-response curve of DOX induced cell death in AC16 cardiomyocytes (CMs) determined by LDH assay. AC16 CMs were treated for 12 hours with different concentrations of doxorubicin (DOX), ranging from 0 to 5 µM. LDH release was determined after 12 hours, and absorbance was measured at 490 nm. Two biological replicates were used, with three technical replicates for each biological replicate.

Supplements Table S1: List of reagents used

**Reagent**	**Manufacturer**	**Catalog Number**	**Origin**
AC16 cardiomyocytes	Millipore	SCC109	Darmstadt, Germany
Dulbecco's Modified Eagle's Medium	Thermo Fisher Scientific	11330-032	Grand Island, NY, USA
Fetal Bovine Serum	Thermo Fisher Scientific	10270-106	Rockford, IL, USA
Antibiotic Antimycotic Solution	Sigma	A5955	Darmstadt, Germany
HyClone™ Trypsin	Fisher Scientific AS	10693313	Oslo, Norway
M-PER	Thermo Fisher Scientific	78501	Rockford, IL, USA

pReceiver-M12 expression-vector	GeneCopoeia	EX-Z0686-M12	Rockville, MD, USA
Control vector pReceiver-M12 expression-vector	GeneCopoeia	EX-EGFP-M12	Rockville, MD, USA
PolyFect Transfection Reagent	Qiagen	301107	Hilden, Germany
Halt™ Phosphatase Inhibitor Cocktail	Thermo Fisher Scientific	78420	Rockford, IL, USA
Halt™ Protease Inhibitor Cocktail	Thermo Fisher Scientific	87786	Rockford, IL, USA
Doxorubicin hydrochloride	Sigma-Aldrich	44583	Saint Louis, MO, USA
Pierce Coomassie Plus Bradford Assay Kit	Thermo Fisher Scientific	23236	Rockford, IL, USA
Caspase-3 Substrate	Merck	235400	Darmstadt, Germany
Caspase-3 Inhibitor	Merck	235423-M	Darmstadt, Germany
2x Laemmli Sample Buffer	Santa Cruz Biotechnology	SC-286962	Dallas, TX, USA
MagicMark™ XP Western Protein Standard	Invitrogen	LC5602	Carlsbad, CA, USA
Precision Plus Protein All Blue Prestained Protein Standard	Bio-Rad	1610373	USA
SuperSignal™ West Pico PLUS Chemiluminescent Substrate	Thermo Fisher Scientific	34580	Rockford, IL, USA
CytoTox 96® Non-Radioactive Cytotoxicity Assay kit	Promega	G1780	Madison, WI, USA
Cell Proliferation Kit I (MTT)	Sigma Aldrich / Roche	11465007001	Mannheim, Germany
DYKDDDDK (FLAG) Tag Monoclonal Antibody (FG4R)	Thermo Fisher Scientific	MA1-91878	USA
β-Actin Antibody (C4)	Santa Cruz Biotechnology	sc-47778	Dallas, TX, USA
Phospho-Akt (Ser473) Antibody	Cell Signaling Technology	9271	Danvers, MA, USA
Akt (pan) (C67E7) Rabbit mAb	Cell Signaling Technology	4691	Danvers, MA, USA

Phospho-GSK-3-beta (Ser9) (D3A4) Rabbit mAb	Cell Signaling Technology	9322	Danvers, MA, USA
GSK-3β (27C10) Rabbit mAb	Cell Signaling Technology	9315	Danvers, MA, USA
**Reagent**	**Manufacturer**	**Catalog Number**	**Origin**
Phospho-p44/42 MAPK (Erk1/2) (Thr202/Tyr204) Antibody	Cell Signaling Technology	9101	Danvers, MA, USA
p44/42 MAPK (Erk1/2) Antibody	Cell Signaling Technology	9102	Danvers, MA, USA
Phospho-Stat3 (Ser727) Antibody	Cell Signaling Technology	9134	Danvers, MA, USA
Stat3 (124H6) Mouse mAb	Cell Signaling Technology	9139	Danvers, MA, USA
Bcl-xL (54H6) Rabbit mAb	Cell Signaling Technology	2764	Danvers, MA, USA
Cyclin D1 Recombinant Rabbit Monoclonal Antibody	Thermo Fisher Scientific	MA5-14512	Waltham, MA, USA
SOD2 Recombinant Rabbit Monoclonal Antibody	Thermo Fisher Scientific	MA5-29578	Waltham, MA, USA
Goat anti Mouse IgG (H/L):HRP	Bio-Rad	STAR207P	Hercules, CA, USA
Goat anti Rabbit IgG (H/L):HRP	Bio-Rad	STAR124P	Hercules, CA, USA
